# Redetermination of [EuCl_2_(H_2_O)_6_]Cl

**DOI:** 10.1107/S1600536814010307

**Published:** 2014-05-10

**Authors:** Frank Tambornino, Philipp Bielec, Constantin Hoch

**Affiliations:** a Ludwig-Maximilians-Universität München, Butenandtstrasse 5-13, D-81377 München, Germany

## Abstract

The crystal structure of the title compound, hexa­aqua­dichlorido­europium(III) chloride, was redetermined with modern crystallographic methods. In comparison with the previous study [Lepert *et al.* (1983 ▶). *Aust. J. Chem.* **36**, 477–482], it could be shown that the atomic coordinates of some O atoms had been confused and now were corrected. Moreover, it was possible to freely refine the positions of the H atoms and thus to improve the accurracy of the crystal structure. [EuCl_2_(H_2_O)_6_]Cl crystallizes with the GdCl_3_·6H_2_O structure-type, exhibiting discrete [EuCl_2_(H_2_O)_6_]^+^ cations as the main building blocks. The main blocks are linked with isolated chloride anions *via* O—H⋯Cl hydrogen bonds into a three-dimensional framework. The Eu^3+^ cation is located on a twofold rotation axis and is coordinated in the form of a Cl_2_O_6_ square anti­prism. One chloride anion coordinates directly to Eu^3+^, whereas the other chloride anion, situated on a twofold rotation axis, is hydrogen bonded to six octa­hedrally arranged water mol­ecules.

## Related literature   

For previous structure determinations of the title compound, see: Lepert *et al.* (1983 ▶); Bel’skii & Struchkov (1965 ▶). For the GdCl_3_·6H_2_O structure type and isotypic compounds, see: Marezio *et al.* (1961 ▶); Bell & Smith (1990 ▶); Burns & Peterson (1971 ▶); Graeber *et al.* (1966 ▶); Habenschuss & Spedding (1980 ▶); Hoch & Simon (2008 ▶); Junk *et al.* (1999 ▶); Reuter *et al.* (1994 ▶). For related structures, see: Demyanets *et al.* (1974 ▶); Reuter *et al.* (1994 ▶). For standardization of crystal data, see: Gelato & Parthé (1987 ▶).

## Experimental   

### 

#### Crystal data  

[EuCl_2_(H_2_O)_6_]Cl
*M* *_r_* = 366.41
Monoclinic, 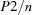

*a* = 9.6438 (12) Å
*b* = 6.5322 (10) Å
*c* = 7.929 (3) Å
β = 93.653 (13)°
*V* = 498.4 (2) Å^3^
*Z* = 2
Ag *K*α radiation
λ = 0.56083 Å
μ = 3.74 mm^−1^
*T* = 293 K
0.23 × 0.20 × 0.18 mm

#### Data collection  

Stoe IPDS I diffractometer
Absorption correction: multi-scan (*MulScanAbs* in *PLATON*; Spek, 2009 ▶) *T*_min_ = 0.425, *T*_max_ = 0.510
13401 measured reflections
1762 independent reflections
1653 reflections with *I* > 2σ(*I*)
*R*_int_ = 0.043

#### Refinement  

*R*[*F* ^2^ > 2σ(*F* ^2^)] = 0.015
*wR*(*F* ^2^) = 0.032
*S* = 1.03
1762 reflections
66 parameters
All H-atom parameters refined
Δρ_max_ = 0.63 e Å^−3^
Δρ_min_ = −0.77 e Å^−3^

### 

Data collection: *X-AREA* (Stoe & Cie, 2006 ▶); cell refinement: *X-AREA*; data reduction: *X-AREA*; program(s) used to solve structure: *SHELXS97* (Sheldrick, 2008 ▶); program(s) used to refine structure: *SHELXL97* (Sheldrick, 2008 ▶); molecular graphics: *DIAMOND* (Crystal Impact, 2007 ▶); software used to prepare material for publication: *SHELXL97*.

## Supplementary Material

Crystal structure: contains datablock(s) global, I. DOI: 10.1107/S1600536814010307/wm5012sup1.cif

Structure factors: contains datablock(s) I. DOI: 10.1107/S1600536814010307/wm5012Isup2.hkl

CCDC reference: 1001456

Additional supporting information:  crystallographic information; 3D view; checkCIF report

## Figures and Tables

**Table 1:** Selected bond lengths (Å)

Eu1—O1	2.4618 (15)
Eu1—O2^i^	2.4620 (18)
Eu1—O3	2.3078 (16)
Eu1—Cl1^ii^	2.7690 (12)

Symmetry codes: (i) 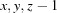; (ii) 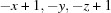.

**Table 2:** Hydrogen-bond geometry (Å, °)

*D*—H⋯*A*	*D*—H	H⋯*A*	*D*⋯*A*	*D*—H⋯*A*
O1—H1⋯Cl2^i^	0.74 (4)	2.36 (4)	3.081 (2)	166.08
O2—H2⋯Cl1^iii^	0.81 (3)	2.54 (3)	3.351 (2)	174.97
O2—H3⋯Cl2^iv^	0.76 (4)	2.51 (4)	3.2234 (19)	157.37
O3—H4⋯Cl1^i^	0.72 (4)	2.35 (4)	3.036 (2)	160.44
O1—H5^v^⋯Cl1	0.74 (2)	2.36 (3)	3.095 (2)	173.89
O3—H6⋯Cl2^vi^	0.79 (4)	2.53 (4)	3.310 (2)	170.66

Symmetry codes: (i) 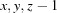; (iii) 
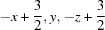
; (iv) 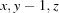; (v) 
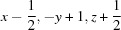
; (vi) 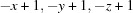.

**Fig. 1.**
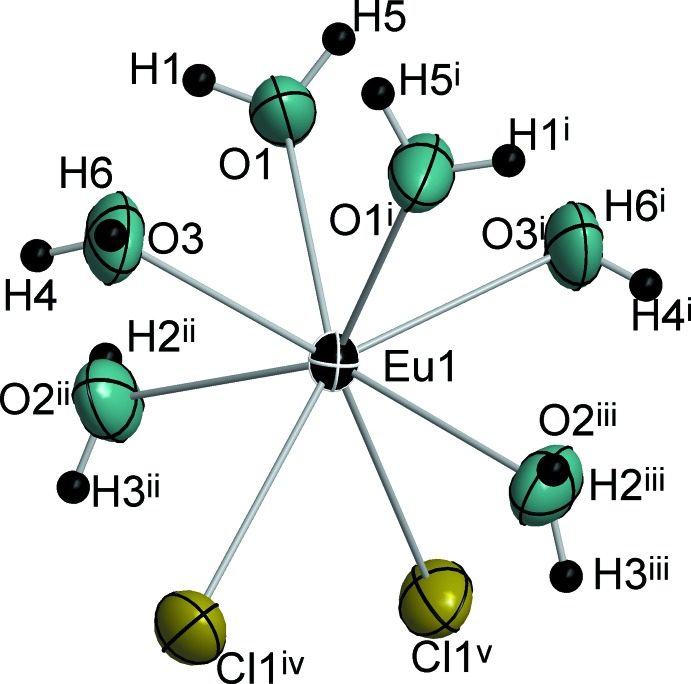
The cationic [Eu(H2O)6Cl2]+ unit in [Eu(H2O)6Cl2]Cl. Ellipsoids are drawn at 75% probability level. Hydrogen atoms are drawn as small black spheres with arbitrary radius. [Symmetry code: (i) 3/2 - x, y, 1/2 - z; (ii) x, y, -1 + z; (iii) 3/2 - x, y, 3/2 - z; (iv) 1 - x, -y, 1 - z; (v) 1/2 + x, -y, -1/2 + z.]

**Fig. 2.**
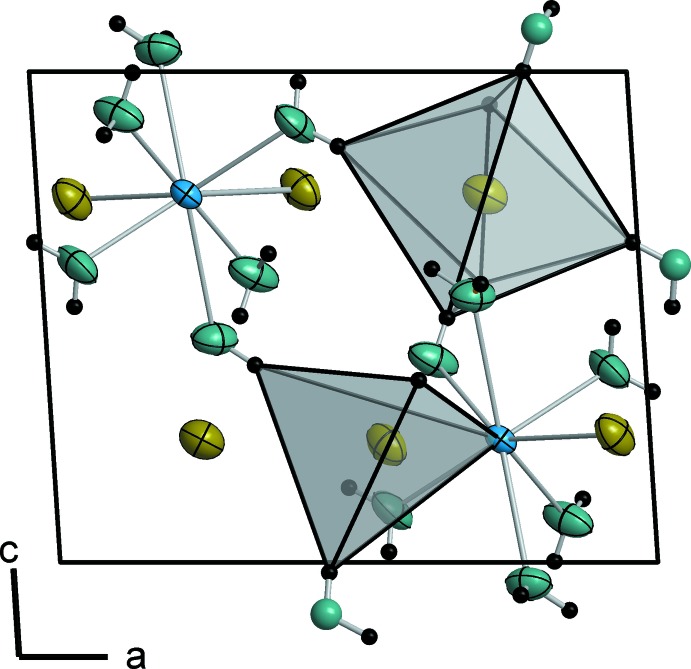
View along [010] on the crystal structure of [Eu(H2O)6Cl2]Cl. Small black spheres represent H atoms, blue ellipsoids represent Eu atoms, olive ellipsoids represent Cl atoms, turquoise ellipsoids represent O atoms. Grey polyhedra represent the coordination of H atoms around Cl atoms.

**Fig. 3.**
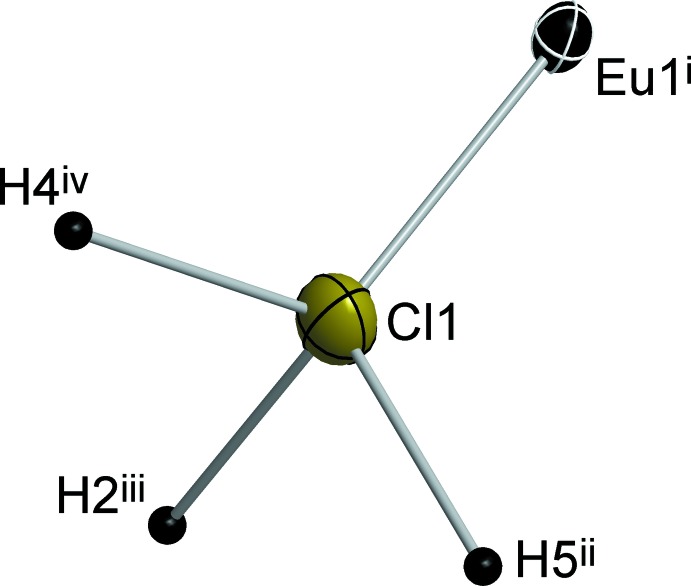
The coordination sphere of the coordinating Cl1 atom is a distorted tetrahedron built from three water molecules and one europium atom. The water molecules coordinate via hydrogen bonds. [Symmetry codes: (i) 1 - x, -y, 1 - z; (ii) 1/2 + x, 1 - y, 1/2 + z; (iii) 3/2 - x, y 3/2 - z; (iv) x, y, 1 + z.]

**Fig. 4.**
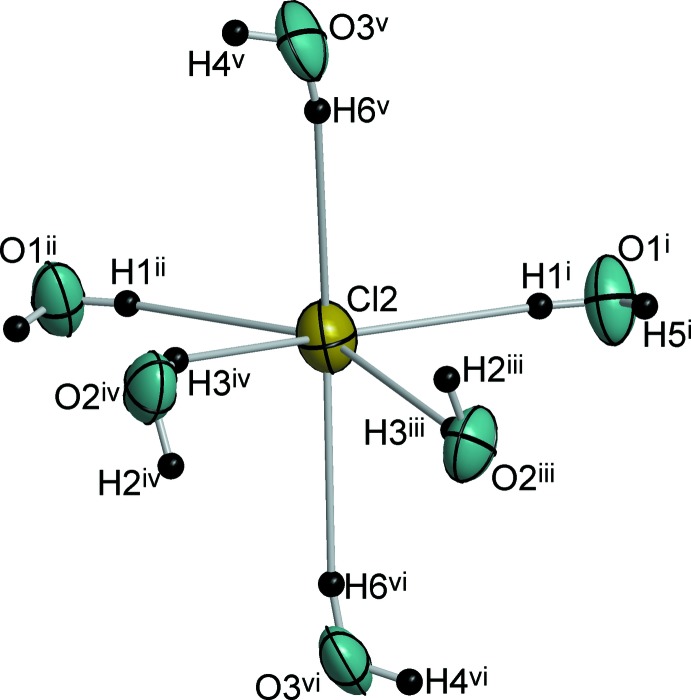
The coordination sphere of the anionic Cl2 atom consists of six water molecules coordinating via their hydrogen atoms forming a distorted octahedron. [Symmetry codes: (i) 3/2 - x, y, 1/2 - z; (ii) x, y, 1 + z; (iii) 3/2 - x, 1 + y, 3/2 - z; (iv) x, 1 + y, z; (v) 1 - x, 1 - y, 1 - z; (vi) 1/2 + x, 1 - y, 1/2 + z.]

## supplementary crystallographic information

### 1. Comment

[EuCl_2_(H_2_O)_6_]Cl crystallizes with the GdCl_3_^.^6H_2_O structure type (Marezio *et al.*, 1961), like many metal trichloride hexahydrates *M*Cl_3_^.^6H_2_O with *M* = Y (Bell & Smith, 1990), Ce (Reuter *et al.*, 1994), Nd (Habenschuss & Spedding, 1980), Sm - Tm (Graeber *et al.*, 1966), Am, Bk (Burns & Peterson, 1971), and three bromide hexahydrates *M*Br_3_^.^6H_2_O with *M* = Pr, Dy (Junk *et al.*, 1999) and Eu (Hoch & Simon, 2008). The first structure determination of the title compound was performed on the basis of film data (Bel'skii & Struchkov, 1965) and without determination of the hydrogen atom positions. A first exact structure determination with all atomic positions was performed by Lepert *et al.* (1983). However, the published data contain errors in the atomic coordinates. We have thus redetermined the structure on the basis of modern area detector data.

The Eu^3+^ cation in [EuCl_2_(H_2_O)_6_]Cl is located on a twofold rotation axis and is coordinated in form of a distorted square antiprism defined by six water molecules and two chloride anions (Fig. 1, Table 1). Hydrogen bonds O—H···Cl connect the [EuCl_2_(H_2_O)_6_]^+^ cations with the Cl^-^ counter-anions to a three-dimensional framework (Fig 2). The complexing chloride anion Cl1 is surrounded by three, the isolated chloride anion Cl2 by six H atoms (Figs. 3, 4), forming hydrogen bonds with Cl···H distances between 2.36 (4) and 2.54 (3) Å (Table 2) and are in good agreement with those in other chloride hydrates. The Eu^III^—O distances in [EuCl_2_(H_2_O)_6_]Cl range from 2.3078 (16) to 2.4620 (18) Å and are comparable with those in EuCl_3_^.^3H_2_O (2.39–2.40 Å; Reuter *et al.*, 1994), EuCl_3_^.^6H_2_O (2.39–2.43 Å; Graeber *et al.*, 1966), or EuCl(OH)_2_ (2.35–2.44 Å; Demyanets *et al.*, 1974) and also with those in EuBr_3_^.^6H_2_O (Hoch & Simon, 2008).

### 2. Experimental

The title compound was obtained by adding small portions of commercially available Eu_2_O_3_ (Alfa Aesar, 99.99%) into concentrated aqueous HCl solution at 353 K until only minor amounts of undissolved Eu_2_O_3_ remained visible for several minutes. The surplus Eu_2_O_3_ finally was dissolved by dropwise addition of concentrated HCl to the solution until a clear colourless solution was obtained. The solution was allowed to cool to 293 K, yielding colourless single-crystal blocks of [EuCl_2_(H_2_O)_6_]Cl.

### 3. Refinement

The positions of all hydrogen atoms were identified from the difference Fourier map and were freely refined, applying one common isotropic displacement parameter to all six H atoms.

For better comparability of our structure model with the previous model by Lepert *et al.* (1983) we haved used the same setting in space group *P*2/*n*. In the crystal structure description given by Lepert *et al.* (1983) several misspellings of the atomic positions were adopted into the databases. The published model leads to diverging refinements if taken as starting values. We have analysed the misspellings and give a conclusive assignment of the atomic positions. If standardized by the program *STRUCTURE-TIDY* (Gelato & Parthé, 1987), the comparison of our model with the one given by Lepert *et al.* (1983) shows, in addition to an origin shift of (0, 1/2, 0), that the *y* and *z* coordinates of atoms O1, O2 and O3 were permutated. In fact, *y*(O1) and *z*(O1) belong to *y*(O3) and *z*(O3), *y*(O2) and *z*(O2) belong to *y*(O1) and *z*(O1), and finally *y*(O3) and *z*(O3) belong to *y*(O2) and *z*(O2). If re-ordered in the given way, the refinement based on starting values from Lepert *et al.* (1983) lead to convergence in few cycles with satisfying results.

### Crystal data

**Table:**

[EuCl_2_(H_2_O)_6_]Cl	*Z* = 2
*M**_r_* = 366.41	*F*(000) = 348
Monoclinic, *P*2/*n*	*D*_x_ = 2.441 Mg m^−^^3^
Hall symbol: -P 2yac	Ag *K*α radiation, λ = 0.56083 Å
*a* = 9.6438 (12) Å	Cell parameters from 13548 reflections
*b* = 6.5322 (10) Å	µ = 3.74 mm^−^^1^
*c* = 7.929 (3) Å	*T* = 293 K
β = 93.653 (13)°	Stretched cuboid, clear colourless
*V* = 498.4 (2) Å^3^	0.23 × 0.20 × 0.18 mm

### Data collection

**Table:**

Stoe IPDS I diffractometer	1762 independent reflections
Radiation source: fine-focus sealed tube	1653 reflections with *I* > 2σ(*I*)
Graphite monochromator	*R*_int_ = 0.043
φ scan	θ_max_ = 25.5°, θ_min_ = 3.0°
Absorption correction: multi-scan (*MulScanAbs* in *PLATON*; Spek, 2009)	*h* = −14→14
*T*_min_ = 0.425, *T*_max_ = 0.510	*k* = −10→10
13401 measured reflections	*l* = −11→11

### Refinement

**Table:**

Refinement on *F*^2^	Primary atom site location: structure-invariant direct methods
Least-squares matrix: full	Secondary atom site location: difference Fourier map
*R*[*F*^2^ > 2σ(*F*^2^)] = 0.015	Hydrogen site location: inferred from neighbouring sites
*wR*(*F*^2^) = 0.032	All H-atom parameters refined
*S* = 1.03	*w* = 1/[σ^2^(*F*_o_^2^) + (0.015*P*)^2^] where *P* = (*F*_o_^2^ + 2*F*_c_^2^)/3
1762 reflections	(Δ/σ)_max_ < 0.001
66 parameters	Δρ_max_ = 0.63 e Å^−^^3^
0 restraints	Δρ_min_ = −0.77 e Å^−^^3^

### Special details

**Table:**

Geometry. All e.s.d.'s (except the e.s.d. in the dihedral angle between two l.s. planes) are estimated using the full covariance matrix. The cell e.s.d.'s are taken into account individually in the estimation of e.s.d.'s in distances, angles and torsion angles; correlations between e.s.d.'s in cell parameters are only used when they are defined by crystal symmetry. An approximate (isotropic) treatment of cell e.s.d.'s is used for estimating e.s.d.'s involving l.s. planes.
Refinement. Refinement of *F*^2^ against ALL reflections. The weighted *R*-factor *wR* and goodness of fit *S* are based on *F*^2^, conventional *R*-factors *R* are based on *F*, with *F* set to zero for negative *F*^2^. The threshold expression of *F*^2^ > σ(*F*^2^) is used only for calculating *R*-factors(gt) *etc*. and is not relevant to the choice of reflections for refinement. *R*-factors based on *F*^2^ are statistically about twice as large as those based on *F*, and *R*- factors based on ALL data will be even larger.

### Fractional atomic coordinates and isotropic or equivalent isotropic displacement parameters (Å^2^)

**Table:**

	*x*	*y*	*z*	*U*_iso_*/*U*_eq_	
Eu1	0.7500	0.150918 (18)	0.2500	0.01345 (3)	
Cl1	0.44156 (5)	0.16532 (7)	0.76010 (6)	0.02588 (9)	
Cl2	0.7500	0.62387 (11)	0.7500	0.02813 (13)	
O1	0.85427 (18)	0.4256 (2)	0.0872 (2)	0.0275 (3)	
O2	0.78164 (18)	0.0484 (2)	0.9561 (2)	0.0263 (3)	
O3	0.56055 (17)	0.3002 (2)	0.1060 (2)	0.0278 (3)	
H1	0.827 (4)	0.454 (6)	0.001 (5)	0.051 (4)*	
H2	0.846 (3)	0.084 (5)	0.902 (4)	0.035 (3)*	
H3	0.766 (4)	−0.063 (7)	0.933 (5)	0.058 (5)*	
H4	0.551 (4)	0.265 (6)	0.020 (5)	0.052 (5)*	
H5	0.881 (4)	0.520 (5)	0.129 (5)	0.040 (3)*	
H6	0.491 (4)	0.319 (6)	0.152 (5)	0.044 (4)*	

### Atomic displacement parameters (Å^2^)

**Table:**

	*U*^11^	*U*^22^	*U*^33^	*U*^12^	*U*^13^	*U*^23^
Eu1	0.01398 (5)	0.01346 (5)	0.01244 (6)	0.000	−0.00293 (3)	0.000
Cl1	0.02417 (18)	0.02396 (18)	0.0286 (2)	−0.00652 (16)	−0.00541 (16)	0.00198 (17)
Cl2	0.0297 (3)	0.0305 (3)	0.0235 (3)	0.000	−0.0033 (2)	0.000
O1	0.0368 (8)	0.0229 (6)	0.0214 (8)	−0.0100 (6)	−0.0085 (6)	0.0038 (5)
O2	0.0336 (7)	0.0277 (7)	0.0175 (7)	−0.0047 (6)	−0.0001 (6)	−0.0039 (5)
O3	0.0250 (6)	0.0317 (7)	0.0250 (8)	0.0067 (5)	−0.0113 (6)	−0.0028 (5)

### Geometric parameters (Å, º)

**Table:**

Eu1—O1	2.4618 (15)	O2—H3	0.76 (4)
Eu1—O1^i^	2.4618 (16)	O2—H2	0.81 (3)
Eu1—O2^ii^	2.4620 (18)	O3—H4	0.72 (4)
Eu1—O2^iii^	2.4620 (18)	O3—H6	0.79 (4)
Eu1—O3	2.3078 (16)	Cl1—H2	2.535 (4)
Eu1—O3^i^	2.3078 (15)	Cl1—H4	2.3535 (4)
Eu1—Cl1^iv^	2.7690 (12)	Cl1—H5^vi^	2.36 (3)
Eu1—Cl1^v^	2.7690 (12)	Cl2—H1^i^	2.36 (4)
O1—H1	0.74 (4)	Cl2—H3^vii^	2.5071 (4)
O1—H5	0.74 (4)	Cl2—H6^viii^	2.53 (4)
			
Eu1—O1—H1	122 (3)	O1—Eu1—O2^ii^	67.83 (6)
Eu1—O1—H1	122 (3)	O1^i^—Eu1—Cl1^iv^	105.35 (5)
Eu1—O1—H5	121 (3)	O1—Eu1—Cl1^iv^	145.35 (4)
Eu1—O1—H5	121 (3)	O2^ii^—Eu1—O2^iii^	148.45 (8)
Eu1^ix^—O2—H2	124 (3)	O2^ii^—Eu1—Cl1^iv^	83.83 (4)
Eu1^ix^—O2—H2	124 (3)	O2^iii^—Eu1—Cl1^iv^	72.65 (4)
Eu1^ix^—O2—H3	117 (3)	O3^i^—Eu1—O1^i^	76.70 (6)
Eu1^ix^—O2—H3	117 (3)	O3—Eu1—O1^i^	67.31 (6)
Eu1—O3—H4	112 (3)	O3^i^—Eu1—O2^ii^	116.15 (7)
Eu1—O3—H4	112 (3)	O3—Eu1—O2^ii^	77.82 (6)
Eu1—O3—H6	120 (3)	O3^i^—Eu1—O3	130.01 (8)
Eu1—O3—H6	120 (3)	O3^i^—Eu1—Cl1^iv^	146.64 (4)
O1^i^—Eu1—O1	86.43 (9)	O3—Eu1—Cl1^iv^	78.18 (5)
O1^i^—Eu1—O2^ii^	140.68 (5)	Cl1^iv^—Eu1—Cl1^v^	83.51 (2)

Symmetry codes: (i) −*x*+3/2, *y*, −*z*+1/2; (ii) *x*, *y*, *z*−1; (iii) −*x*+3/2, *y*, −*z*+3/2; (iv) −*x*+1, −*y*, −*z*+1; (v) *x*+1/2, −*y*, *z*−1/2; (vi) *x*−1/2, −*y*+1, *z*+1/2; (vii) *x*, *y*+1, *z*; (viii) −*x*+1, −*y*+1, −*z*+1; (ix) *x*, *y*, *z*+1.

### Hydrogen-bond geometry (Å, º)

**Table:**

*D*—H···*A*	*D*—H	H···*A*	*D*···*A*	*D*—H···*A*
O1—H1···Cl2^ii^	0.74 (4)	2.36 (4)	3.081 (2)	166.08
O2—H2···Cl1^iii^	0.81 (3)	2.54 (3)	3.351 (2)	174.97
O2—H3···Cl2^x^	0.76 (4)	2.51 (4)	3.2234 (19)	157.37
O3—H4···Cl1^ii^	0.72 (4)	2.35 (4)	3.036 (2)	160.44
O1—H5^vi^···Cl1	0.74 (2)	2.36 (3)	3.095 (2)	173.89
O3—H6···Cl2^viii^	0.79 (4)	2.53 (4)	3.310 (2)	170.66

Symmetry codes: (ii) *x*, *y*, *z*−1; (iii) −*x*+3/2, *y*, −*z*+3/2; (vi) *x*−1/2, −*y*+1, *z*+1/2; (viii) −*x*+1, −*y*+1, −*z*+1; (x) *x*, *y*−1, *z*.

## References

[bb1] Bell, A. M. T. & Smith, A. J. (1990). *Acta Cryst.* C**46**, 960–962.

[bb2] Bel’skii, N. K. & Struchkov, Yu. T. (1965). *Kristallografiya*, **10**, 21–28.

[bb3] Burns, J. H. & Peterson, J. R. (1971). *Inorg. Chem.* **10**, 147–151.

[bb4] Crystal Impact (2007). *DIAMOND* Crystal Impact GbR, Bonn, Germany.

[bb5] Demyanets, L. N., Bukin, V. I., Emelyanova, E. N. & Ivanov, V. I. (1974). *Sov. Phys. Crystallogr.* **18**, 806–808.

[bb6] Gelato, L. M. & Parthé, E. (1987). *J. Appl. Cryst.* **20**, 139–143.

[bb7] Graeber, E. J., Conrad, G. H. & Duliere, S. F. (1966). *Acta Cryst.* **21**, 1012–1013.

[bb8] Habenschuss, A. & Spedding, F. H. (1980). *Cryst. Struct. Commun.* **9**, 71–75.

[bb9] Hoch, C. & Simon, A. (2008). *Acta Cryst.* E**64**, i35. 10.1107/S1600536808014359 PMC2961385 21202437

[bb10] Junk, P. C., Semenova, L. I., Skelton, B. W. & White, A. H. (1999). *Aust. J. Chem.* **52**, 531–538.

[bb11] Lepert, D. L., Patrick, J. M. & White, A. H. (1983). *Aust. J. Chem.* **36**, 477–482.

[bb12] Marezio, M., Plettinger, H. A. & Zachariasen, W. H. (1961). *Acta Cryst.* **14**, 234–236.

[bb13] Reuter, G., Fink, H. & Seifert, H. J. (1994). *Z. Anorg. Allg. Chem.* **620**, 665–671.

[bb14] Sheldrick, G. M. (2008). *Acta Cryst.* A**64**, 112–122. 10.1107/S0108767307043930 18156677

[bb15] Spek, A. L. (2009). *Acta Cryst.* D**65**, 148–155. 10.1107/S090744490804362X PMC2631630 19171970

[bb16] Stoe & Cie (2006). *X-AREA* Stoe & Cie GmbH, Darmstadt, Germany.

